# Socio-economic and production dynamics of Guinea fowl farming in Northern Ghana: insights into health management, challenges, and climate change impacts

**DOI:** 10.1007/s11250-025-04427-2

**Published:** 2025-04-22

**Authors:** Nasir Abdallah, Oyebamiji A. Oluwaseun

**Affiliations:** 1 Faculty of Agriculture, Department of Animal Science, Çukurova University, Adana, Türkiye; 2 Department of Agricultural Economics, Ege University, Institute of Natural and Applied Science, Izmir, Türkiye

**Keywords:** Climate change, Ghana, Guinea fowl, Health, Marketing, Production, Q12, Q54, O13, Q18

## Abstract

Guinea fowl farming is vital to the livelihoods of rural communities in northern Ghana, yet its socio-economic and production dynamics remain underexplored, especially concerning health management and climate change. This study bridges this gap by investigating the socio-economic profiles, production practices, and health challenges of guinea fowl farmers in three towns in northern Ghana. A total of 137 farmers participated in structured interviews, with data analyzed using one-way and two-way ANOVA tests in SPSS version 21. The results highlighted variations in farmer demographics, with most being married, involved in crop farming, and having non-formal education. Guinea fowl production served for both sale and home consumption, with adult birds, keets, and young birds priced at > 69, 9–10, and 18–30 Cedis, respectively, while fertile and table eggs cost 4–4.5 Cedis. Flock sizes ranged from 10–60, predominantly of the Lavender breed. Farmers favored semi-intensive systems with traditional poultry shelters, supplemented feed, and pond or river serving as water sources. Disease symptoms, such as wing drooping, and high mortality rates were major challenges, with climate change exacerbating disease prevalence and management costs. These findings highlight the need for enhanced disease management, climate-resilient practices, and targeted interventions to ensure sustainable guinea fowl production and improved livelihoods.

## Introduction

Agriculture plays a pivotal role in reducing poverty and achieving food security, aligning with Sustainable Development Goals (SDGs) 1 and 2, which aim to eradicate poverty and ensure zero hunger, respectively (Abdallah and Oyebamiji 2024). However, achieving these goals is hindered by the adverse effects of climate change which disrupt food production systems and threaten agricultural livelihoods (Adjei and Oyebamiji 2024; Mugambiwa and Tirivangasi 2017). In Africa, the intersection of poverty, food insecurity, and climate change emphasizes the struggle to achieve SDGs 1 and 2 (Oyebamiji and Türkekul 2022; Qasemi et al. 2023). Gil et al. (2019) observed that agriculture contributes to these goals by reducing malnutrition, ensuring nutritious food availability, and supporting overall well-being, further reinforcing its critical role in achieving health-related SDG 3.

Within Ghana’s agricultural sector, the poultry industry has been steadily growing since 2000, contributing significantly to household incomes and providing a vital protein source (Kusi et al. 2015; Adu-Aboagye et al. 2020). Guinea fowl (*Numida meleagris*) is an important component of the poultry subsector in Northern Ghana, where its production is more prevalent compared to other regions (Agbolosu et al. 2012). These birds are mainly raised in backyard systems, often alongside chickens, using extensive or semi-intensive methods, with intensive housing more common in the southern regions (Dei and Karbo 2004; Annor et al. 2012). Guinea fowl production serves as a key source of income, enabling rural households to meet emergency financial needs and improve food security (Baimbill-Johnson et al. 2021). Additionally, guinea fowl meat provides an affordable and protein-rich dietary option, addressing malnutrition risks in rural areas. Its status as a delicacy also drives demand in other parts of the country (Issaka and Yeboah 2016).

The production of guinea fowl plays a critical role in addressing poverty and food security in the Northern Region of Ghana. Despite being one of the poorest regions in the country, farming remains the primary occupation of its inhabitants. A key feature of Ghana’s economy is the disproportionate concentration of poverty in the three northern regions: the Upper East, Upper West, and Northern Regions. Reports by Molini and Paci (2015) indicate that nearly 40% of Ghana’s poor population resides in the north, which accounts for only 17% of the national population. The Northern Region, in particular, is the largest single contributor to poverty in Ghana, with approximately 1.3 million of the country’s poor living there (Ghana Statistical Service 2014). Extreme poverty levels in the north range from 21.3% in the Upper East to 45.1% in the Upper West, far exceeding the national average of 8.4% (Ghana Statistical Service 2014).

Despite the economic and nutritional significance of guinea fowl production, there is limited research on its production dynamics in Ghana, particularly in climate-vulnerable regions such as the Northern Region. The existing literature lacks in-depth exploration of the impacts of climate change on guinea fowl production, especially in areas identified as climate change hotspots. Although guinea fowls are more drought-resistant compared to other poultry species, they are not immune to the broader impacts of climate change. Critical factors such as the effects of climate variability on production and income, as well as the sources of funding for guinea fowl farming, remain underexplored. Climate change influences key production factors, including the cost of production, availability of water and feed, and disease dynamics. To address these gaps, this study investigates guinea fowl production among smallholder farmers in three towns—Dungu, Kula, and Golinga—in the Northern Region. The survey aims to provide a comprehensive understanding of guinea fowl farming practices in these communities while examining key factors that influence production efficiency and sustainability.

## Materials and methods

### Study area

This study was conducted in the Northern Region of Ghana, focusing on three towns: Golinga in the Tolon District, and Dungu and Kula in the Tamale District. These towns were selected not because they are the only locations where guinea fowl farming occurs, but because they are representative of rural communities in the region where guinea fowl production is a significant agricultural activity. The selection was based on the prevalence of guinea fowl farming in these areas, as well as their shared characteristics, such as their rural nature, reliance on crop and livestock farming, and typical vegetation and climatic conditions of the Northern Savannah Zones of Ghana. These towns provide a valuable snapshot of the challenges and opportunities associated with guinea fowl production in the region, reflecting broader patterns observed across similar communities in Northern Ghana. The Tolon District is located in the northwest part of the Northern Region (9°25′51.6″N 1°3′53.64″W). The district experiences a single rainy season, which begins in late April and lasts until October or November. The dry season spans November to March, with ambient temperatures ranging between 33 °C and 39 °C during the day and between 20 °C and 26 °C at night while the average annual rainfall is between 950 mm and 1, 200 mm (Issaka and Yeboah 2016). Grasslands dominate the vegetation, characterized by drought-resistant trees. Additionally, most inhabitants are peasants and subsistence farmers.

**Fig. 1:**
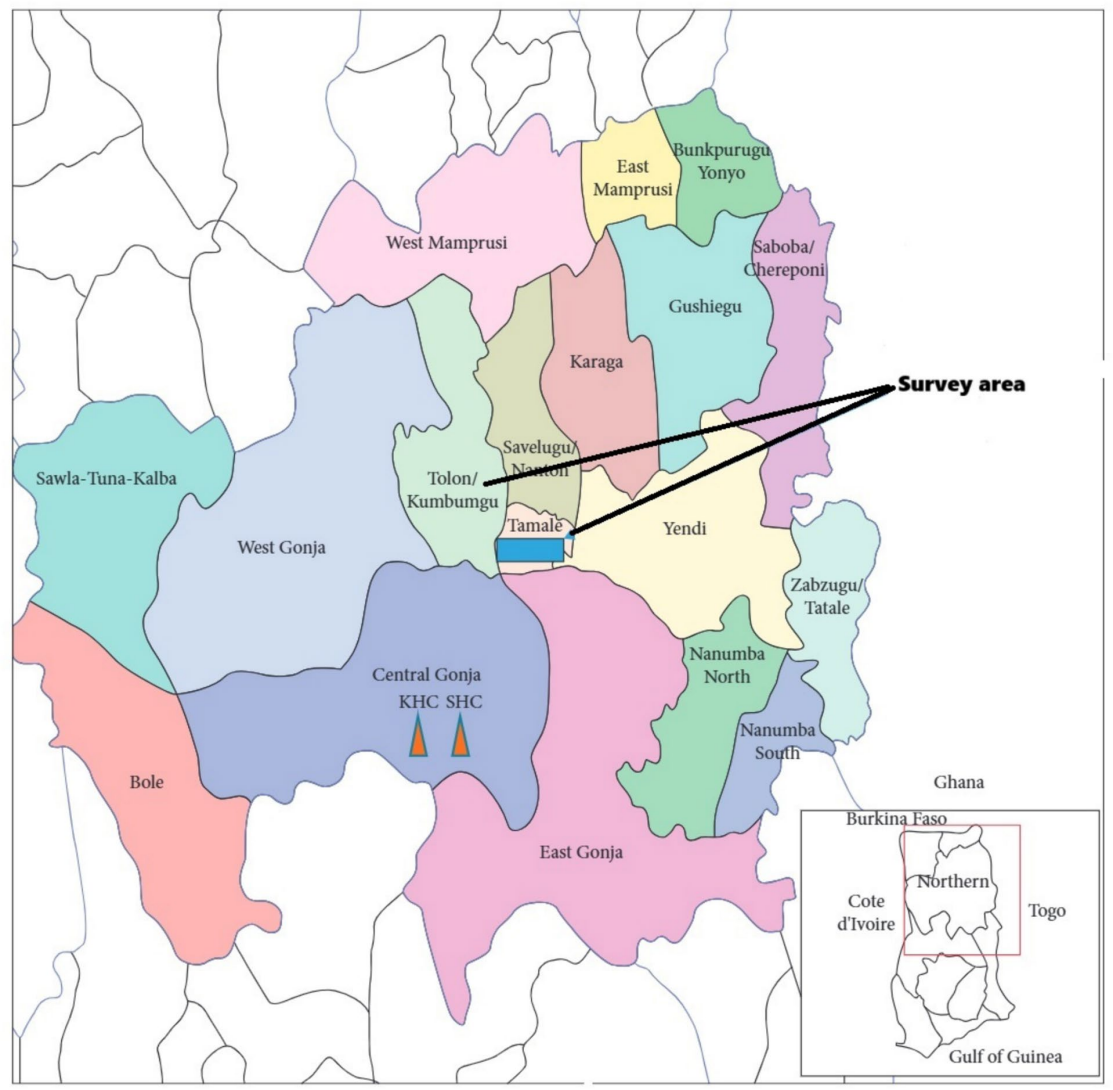
Map of Ghana showing the study area (Tolon and Tamale districts) and neighboring communities and districts (Source; Helegbe et al. 2018)

The Tamale Metropolitan District is also in the northwest part of the Northern Region, with Tamale as its capital (9°24′30.1″N 0°50′25.63″W). The proportion of the population living in urban localities (80.08%) is higher than that in rural areas (19.1%). The district covers an area of about 647 km^2^ and borders the Sagnarigu District to the west and north, the Mion District to the east, the East Gonja District to the south, and the Central Gonja District to the southwest. Most residents are employed in agriculture, along with other sectors such as teaching, manufacturing, and trading. The annual rainfall is approximately 1100 mm and the mean day temperatures range from 28 °C (December and mid-April) to 43 °C (March, early April) while the mean night temperatures range from 18 (December) to 25 °C (February, March). The map of Ghana showing the survey area is given in Fig. 1.

### Sampling

Non-probability snowball sampling, where farmers were selected based on their networks rather than from a database or probability sampling (Koné et al. 2018; Orounladji et al. 2022), was employed. The choice of snowballing in this study was influenced by four factors; In rural settings, trust and personal relationships play a significant role in gaining access to participants, snowballing implies that the first farmer interviewed trusted us and thus do the second referent; given the study’s resources and time constraints, snowball sampling proved to be a practical and efficient method for identifying guinea fowl farmer; and by starting with a few well-informed participants (experienced guinea fowl farmers), the study was able to identify other key stakeholders in the guinea fowl farming community. The first guinea fowl farmer interviewed provided information about other farmers, who in turn referred us to additional farmers they knew (Kouassi et al. 2019; Orounladji et al. 2022). The interviews continued until the majority of guinea fowl farmers in each village were interviewed, or the list of farmers in each village was exhausted. The selection of districts and towns was based on guinea fowl farming activities, as identified by a local agent involved in buying guinea fowl from the north and selling them to customers in other parts of Ghana.

### Data collection

This study was carried out in 2024 between July and October. Primary data was obtained through face-to-face interviews of farmers who reared at least 10 guinea fowls. The questions were first created in an online software, Kobo Toolbox which was then downloaded on an Android Smartphone (Abdallah et al. 2024). The questionnaires consisted of socio-economic characteristics of farmers, marketing, and production characteristics, health management, and climate change-related questions.

### Statistical analysis

Cross-tabulation was used to evaluate the descriptive statistics, and the data were presented in frequencies and percentages. Price-related parameters were evaluated using a one-way ANOVA test, while non-price related parameters were analyzed with a two-way ANOVA. Statistical significance was set at P ≤ 0.05. All analyses were conducted using SPSS version 21. Multiple corresponding analysis was done using R version 4.4.2.

### Multiple correspondence analysis of production characteristics of Guinea fowl farms

Multiple Correspondence Analysis (MCA) is a useful technique for analyzing categorical data and identifying relationships among various socio-economic and production factors related to guinea fowl farming in northern Ghana. MCA is particularly relevant here as it helps uncover patterns and groupings among variables such as farmers'marital status, education, occupation, flock size, and knowledge of climate change, among others.

## Results

The socio-economic characteristics of the guinea fowl farmers interviewed are shown in Table 1. The age distribution of farmers varied significantly among the communities (*P* < 0.05). Most farmers in Dungu were aged 30–41, while the majority in Golinga and Kula were aged 42–55 and 18–29, respectively. Across all towns, farmers aged 56 years and above represented the smallest group.

**Table 1:** Socio-economic characteristics of interviewed guinea fowl farmers in the study area

Character	Modality	Study area						Mean square	Probability
		Dungu (*n* = 51)		Golinga (*n* = 36)		Kula (*n* = 50)			
		*n*	*%*	*n*	*%*	*n*	*%*		
Age	18–29	13	25.49	7	19.44	21	42.00	1689.67	< 0.001
	30–41	17	33.33	9	25.00	16	32.00		
	42–55	11	21.57	13	36.11	10	20.00		
	56 and above	10	19.61	7	19.44	3	6.00		
Marital status	Single	9	17.65	4	11.11	10	20.00	1101.70	0.002
	Married	35	68.63	25	69.44	37	74.00		
	Divorced	0	0.00	1	2.78	0	0.00		
	Widow (er)	7	13.73	6	16.67	3	6.00		
Education	Primary	12	23.53	9	25.00	17	34.00	931.02	0.003
	Secondary	11	21.57	3	8.33	7	14.00		
	Tertiary	1	1.96	3	8.33	0	0.00		
	Vocational	0	0.00	1	2.78	0	0.00		
	Non-formal	27	52.94	20	55.56	26	52.00		
Main occupation	Blacksmith	0	0.00	1	2.78	0	0.00	68.94	0.004
	Butcher	1	1.96	3	8.33	0	0.00		
	Crop farming	34	66.67	14	38.89	43	86.00		
	Livestock farming	10	19.61	12	33.33	1	2.00		
	Mechanic	4	7.84	0	0.00	0	0.00		
	Mixed farming	0	0.00	0	0.00	1	2.00		
	Mobile money vendor	0	0.00	1	2.78	0	0.00		
	Student	2	3.92	1	2.78	5	10.00		
	Teacher	0	0.00	3	8.33	0	0.00		
	Vulcanizer	0	0.00	1	2.78	0	0.00		
Training in breeding guinea fowls	Yes	50	98.04	36	100.00	47	94.00	243.34	0.311
	No	1	1.96	0	0.00	3	6.00		
Years of experience in breeding guinea fowls	1–12 years	25	49.02	8	22.22	23	46.00	1182.92	0.001
	13–23 years	10	19.61	12	33.33	18	36.00		
	24–34 years	9	17.65	12	33.33	8	16.00		
	35–45 years	7	13.73	4	11.11	1	2.00		
Sources of finance for guinea fowl production	Self/Personal finance	51	100.00	36	100.00	50	100.00	-	-
	External funding	0	0.00	0	0.00	0	0.00		

*n = number, % = percentage source (Survey 2024)*

The marital status of the farmers also varied significantly among the towns (*P* < 0.05). Kula had the highest number of single and married farmers, while Golinga recorded the highest percentage of divorced and widowed participants. Differences in educational background were statistically significant (*P* < 0.05), with the majority in all towns having non-formal education. Golinga had the highest number of farmers with non-formal education, while Kula and Dungu had the highest numbers of farmers with primary and secondary education, respectively. Farmers with tertiary or vocational education were the least represented in all towns.

The main occupational background of farmers differed significantly among the towns (*P* < 0.05) with crop farming being the primary occupation in all the towns surveyed. Other occupations, such as teaching, mechanics, butchering, and blacksmithing, were observed in smaller percentages across all towns.

Training in guinea fowl breeding did not differ significantly among the towns (*P* > 0.05). However, farmers’ experience in breeding guinea fowl varied significantly (*P* < 0.05), with Dungu having the highest number of farmers with 1–12 years and 35–45 years of experience. In contrast, Kula had the highest percentage of farmers with 13–23 years of experience, and Golinga had the highest percentage with 24–34 years of experience. None of the farmers interviewed reported receiving external funding from government or private sources to support guinea fowl production.

The marketing characteristics of the surveyed guinea fowl farms are presented in Table 2. The reasons for guinea fowl production, major sources of income, price of table eggs, and selling locations did not differ significantly among towns (*P* > 0.05). However, the prices of adult and young guinea fowls, keets, and fertile eggs showed significant variation (*P* < 0.05). Most adult guinea fowls were sold for > 69 Cedis, with the highest and lowest numbers of farmers selling at this price in Dungu and Kula, respectively. The price of keets was typically 18–30 Cedis, with most farmers in Dungu selling within this range. Only a few farmers in Golinga and Kula sold keets at 31–41 or > 41 Cedis. Many farmers in Kula were unaware of keet pricing. Fertile eggs were mainly sold at 4–4.5 Cedis, with most sellers at this price being in Dungu. Only a few farmers in all towns sold fertile eggs for more than 4.5 Cedis.

**Table 2:** Marketing characteristics of guinea fowl farmers in study area

Character	Modality	Study area						Mean square	Probability
		Dungu (*n* = 51)		Golinga (*n* = 36)		Kula (*n* = 50)			
		*n*	*%*	*n*	*%*	*n*	*%*		
Reason for guinea fowl production	Home consumption	0	0.00	0	0.00	0	0.00	11.32	0.825
	Sale	1	1.96	0	0.00	8	16.00		
	Home consumption + Sale	50	98.04	36	100.00	42	84.00		
Major source of income	Selling guinea fowl eggs	51	100.0	36	100.00	43	86.00	11.31	0.526
	Selling live Guinea fowl	0	0.00	0	0.00	7	14.00		
Price of adult guinea fowls (Cedis)	28–38	0	0.00	1	2.77	0	0.00	181.34	0.002
	39–49	0	0.00	0	0.00	1	2.00		
	50–69	0	0.00	0	0.00	8	16.00		
	> 69	51	100.00	35	97.22	41	82.00		
Price of young guinea fowls (Cedis)	18–30	43	84.31	28	77.78	18	36.00	241.27	< 0.001
	31–40	0	0.00	6	16.67	5	10.00		
	> 41	0	0.00	0	0.00	2	4.00		
	No idea	8	15.69	2	5.56	25	50.00		
Price of keets (Cedis)	9–10	39	76.47	23	63.89	10	20.00	2.640	0.049
	11–13	4	7.84	11	30.56	1	2.00		
	No idea	8	15.69	2	5.56	39	78.00		
Price of fertile eggs (Cedis)	4–4.5	45	88.24	30	83.33	43	86.00	104.86	< 0.001
	> 4.5	6	11.76	6	16.67	7	14.00		
Price of table eggs (Cedis)	4.0	6	11.76	10	27.78	48	96.00	61.66	0.608
	4.5	45	88.23	26	72.22	2	4.00		
Selling locations	Market	2	3.92	0	0.00	11	22.00	139.56	0.555
	Home/Farms	8	15.69	0	0.00	4	8.00		
	Market + Home/Farms	41	80.39	36	100.00	35	70.00		

*n = number, % = percentage source (Survey 2024)*

The production characteristics of guinea fowl farms surveyed are presented in Table 3. All farmers in the three towns reared guinea fowls for both their meat and eggs. Additionally, the number of guinea fowls per flock varied significantly among the towns (P < 0.05), with the proportion of farmers characterized by small flock sizes being highest in Golinga and lowest in Kula. Farmers with medium–low flock sizes were more common in Kula and less common in Golinga. The method of production also varied significantly among the towns, with all farmers in Dungu and Golinga using the semi-intensive system; however, few farmers in Kula employed the extensive system (*P* < 0.05). The type of housing or shelter also differed significantly among the towns (*P *< 0.05). While all farmers in Dungu and Golinga used traditional shelters, 2% of farmers in Kula used improved shelters, and 10% did not provide any form of shelter. Feed type was also statistically different (*P* < 0.05) among the towns, with the use of supplements being highest in Kula and lowest in Dungu. The use of both compound feed and supplement was highest in Dungu and lowest in Kula. Additionally, all farmers in the towns provided drinking water to their guinea fowls.

**Table 3:** Production characteristics of guinea fowl farms

Character	Modality	Study area						Mean square	Probability
		Dungu (*n* = *51*)		Golinga (*n* = *36*)		Kula (*n* = *50*)			
		*n*	%	*n*	%	*n*	%		
Purpose of production	Meat	0	0.00	0	0.00	0	0.00	-	-
	Egg	0	0.00	0	0.00	0	0.00		
	Meat + Egg	51	100	36	100.00	50	100.00		
Number of guinea fowls per flock	small flock size (10–30)	29	56.86	21	58.33	18	36.00	8492.37	< 0.001
	Medium–low flock size (31–60)	22	43.14	14	38.89	28	56.00		
	Upper-medium flock (61–90) size	0	0.00	0	0.00	4	8.00		
	High flock size (> 90)	0	0.00	1	2.78	0	0.00		
Guinea fowl breed reared	Lavender	51	100.00	35	97.22	50	100.00	115.21	0.485
	Pearl	0	0.00	1	2.78	0	0.00		
	White	0	0.00	0	0.00	0	0.00		
	Mixed flock	0	0.00	0	0.00	0	0.00		
Predominant guinea fowl age group in flock	Adult Guinea Fowls	51	100.00	36	100.00	49	98.00	74.96	0.575
	Young Guinea Fowls	0	0.00	0	0.00	1	2.00		
	Keets	0	0.00	0	0.00	0	0.00	5703.12	< 0.001
Method of production	Extensive	0	0.00	0	0.00	5	10.00		
	Semi-Intensive	51	10.00	36	100.00	45	90.00		
	Intensive	0	0.00	0	0.00	0	0.00		
Type of housing/Shelter	Traditional Poultry house	51	100.00	36	100.00	44	88.00	2900.67	< 0.001
	Improved/modern house	0	0.00	0	0.00	1	2.00		
	No shelter	0	0.00	0	0.00	5	10.00		
Feed type	Supplements	20	39.22	24	66.67	42	84.00	2195.54	0.002
	Compound feed + supplements	31	60.78	12	33.33	8	16.00		
	No feed	0	0.00	0	0.00	0	0.00		
	Compound feed	0	0.00	0	0.00	0	0.00		
Type of compound feed used	Broiler feed	2	6.45	3	25.00	1	12.50	431.98	0.125
	Commercial guinea fowl feed	28	90.32	9	75.00	0	0.00		
	Broiler + laying hen feed	1	3.23	0	0.00	7	87.50		
	Laying hen feed	0	0.00	0	0.00	0	0.00		
Type of supplements used the most	Maize	16	31.37	16	44.44	25	50.00	312.76	0.254
	Millet	15	29.41	14	38.89	18	36.00		
	Sorghum	19	37.25	6	16.67	7	14.00		
	Kitchen waste	1	1.96	0	0.00	0	0.00		
	Rice	0	0.00	0	0.00	0	0.00		
	Termites	0	0.00	0	0.00	0	0.00		
Provision of drinking water to guinea fowls	Yes	51	100.00	36	100.00	50	100.00	-	-
	No	0	0.00	0	0.00	0	0.00		
Sources of drinking water for guinea fowls	Pond/well	27	52.94	7	19.44	0	0.00	492.26	0.125
	River	8	15.69	23	63.89	50	100.00		
	Tap	16	31.37	6	16.67	0	0.00		

*n = number, % = percentage Source (survey, 2024)*

The type of guinea fowl breeds reared, the predominant age group in flocks, the type of compound feed, the most frequently used supplement, and the sources of drinking water for guinea fowls did not differ significantly among the towns (*P* > 0.05).

The health management characteristics of the farms surveyed are summarized in Table 4. Symptoms of guinea fowl diseases, prophylactic measures, frequency of vaccination, type of treatment, personnel treating sick birds, percentage of keet and adult guinea fowl mortality, and constraints to keet production were statistically insignificant among the towns (*P* > 0.05). However, constraints to adult guinea fowl production differed significantly (*P* < 0.05). Losses due to predation were highest in Golinga, while taming of birds and diseases were the major constraints in Dungu. Additionally, bird losses due to theft were higher in Kula compared to the other towns.

**Table 4:** Characteristics of guinea fowl health management system

Character	Modality	Study area						Mean square	Probability
		Dungu *(n* = *51*)		Golinga (*n* = *36*)		Kula (*n* = *50*)			
		n	%	n	%	n	%		
Symptoms of guinea fowl disease	Wing drooping	43	84.31	27	75.00	23	46.00	429.02	0.155
	Diarrhea	7	13.73	9	25.00	27	54.00		
	Swollen head	1	1.96	0	0.00	0	0.00		
Prophylactic measures	Hygiene + vaccination	50	98.04	36	100.00	50	10.00	17.29	0.787
	None	1	1.96	0	0.00	0	0.00		
	Hygiene	0	0.00	0	0.00	0	0.00		
	Vaccination	0	0.00	0	0.00	0	0.00		
Frequency of vaccination	1 time per year	49	98.00	36	100.00	49	98.00	10.61	0.833
	> 1 time per year	1	2.00	0	0.00	1	2.00		
Type of treatment used	Modern	0	0.00	1	2.78	0	0.00	611.91	0.073
	Traditional	0	0.00	0	0.00	5	10		
	Modern + traditional	50	100.00	35	97.22	45	90		
Person treating sick birds	Veterinarian	1	1.96	0	0.00	0	0.00	49.18	0.814
	Farmer	48	94.12	31	86.11	48	96.00		
	Farmer + Veterinarian	2	3.92	5	13.89	2	4.00		
Major constraint to keets production	High keet mortality	51	100.00	36	100.00	46	92.00	72.79	0.735
	Predation	0	0.00	0	0.00	1	2.00		
	Lack of quality hatching eggs	0	0.00	0	0.00	3	6.00		
	Housing	0	0.00	0	0.00	0	0.00		
	Disease	0	0.00	0	0.00	0	0.00		
	Theft	0	0.00	0	0.00	0	0.00		
	Nutrition	0	0.00	0	0.00	0	0.00		
Percentage of keets mortality	< 20	0	0.00	0	0.00	3	6.00	80.26	0.714
	20–50	5	9.80	9	25.00	32	64.00		
	> 50	46	90.20	27	75.00	15	30.00		
Constraint to adult guinea fowl production	High cost of production	1	1.96	1	2.78	5	10.00	842.33	< 0.001
	High mortality	0	0.00	3	8.33	3	6.00		
	Predation	11	21.57	15	41.67	16	32.00		
	Taming of birds	14	27.45	6	16.67	2	4.00		
	Housing	0	0.00	1	2.78	5	10.00		
	Disease	13	25.49	5	13.89	1	2.00		
	Theft	11	21.57	4	11.11	14	28.00		
	Nutrition	0	0.00	1	2.78	4	8.00		
	Poor productivity of birds	1	1.96	0	0.00	0	0.00		
Percentage of Adult guinea fowl mortality	< 20	14	27.45	5	13.89	44	88.00	337.54	0.221
	20–50	34	66.67	30	83.33	5	10.00		
	> 50	3	5.88	1	2.78	1	2.00		

*n = number, % = percentage Source (Survey, 2024)*

Table 5 summarizes farmers’ knowledge of climate change and its impact on guinea fowl production. All farmers in the three towns were aware of climate change and the knowledge of climate change varied significantly among the towns (*P* < 0.05). The proportion of farmers with"average"knowledge of climate change was highest in Dungu and lowest in Golinga. Conversely, the proportion of farmers with"good"knowledge of climate change was highest in Kula and lowest in Dungu. Specific changes due to climate change, its profound effects, income changes, and additional costs attributed to climate change were statistically insignificant among the towns (*P* > 0.05).

**Table 5:** Awareness of climate change and its impact on guinea fowl farming

Character	Modality	Study area						Mean square	Probability
		Dungu (*n* = 51)		Golinga (*n* = 36)		Kula (*n* = 50)			
		*n*	*%*	*n*	*%*	*n*	*%*		
Awareness of climate change and its impacts	Yes	51	100.00	36	100.00	50	100.00	-	-
	No	0	0.00	0	0.00	0	0.00		
Self-rated knowledge of climate change	1 (Below average)	0	0.00	0	0.00	0	0.00	2378.16	< 0.001
	2 (Average)	18	35.29	7	19.44	11	22.00		
	3 (Good)	29	56.86	22	61.11	33	66.00		
	4 (Very good)	4	7.84	7	19.44	6	12.00		
	5 (Excellent)	0	0.00	0	0.00	0	0.00		
Observed specific variations in environmental conditions due to climate change	Changes in environmental temperature	28	54.90	31	86.11	46	92.00	494.66	0.147
	Changes in rainfall	23	45.10	5	13.89	4	8.00		
	Changes in the pattern of poultry diseases	0	0.00	0	0.00	0	0.00		
Most profound effect of climate change on guinea fowl production	High mortality	0	0.00	0	0.00	9	18.00	686.14	0.087
	High spread of pest and diseases	51	100.00	36	100.00	41	82.00		
	Decrease in production	0	0.00	0	0.00	0	0.00		
	Decrease in the availability of feed and water resources	0	0.00	0	0.00	0	0.00		
Estimated reduction in income due to climate variability	< 20	17	33.33	2	5.56	42	84.00	280.68	0.310
	20–50	33	64.71	33	91.67	8	16.00		
	> 50	0	0.00	1	2.78	0	0.00		
Additional cost incurred due to climate-related issues	Additional cost of feed	1	1.96	0	0.00	0	0.00	0.794	0.954
	Control of pest and disease	50	98.04	36	100.00	50	100.00		
	Additional cost for housing structures	0	0.00	0	0.00	0	0.00		

*n = number, % = percentage source (Survey 2024)*

Table 6 above summarizes the variables, their codes, and respective modalities used in a Multiple Correspondence Analysis (MCA) to investigate factors influencing guinea fowl production. The variables selected for the MCA include important production related factors like the type of feed used, water source, keet mortality rates, constraints to guinea fowl production, and farmers'awareness of climate change. By using MCA, we can explore how these factors interact and how they may influence the production and health dynamics of guinea fowl farming in different towns. The analysis allows us to visualize relationships between production categorical variables, which is crucial for understanding complex socio-economic patterns of guinea fowl farming in the study area. In the context of the study, MCA was used to assess how farmers'socio-economic profiles (e.g., marital status, education level) correlate with production outcomes such as keet mortality or the effectiveness of climate change mitigation strategies. It also aids in identifying potential policy interventions by highlighting key variables that require attention, such as improving disease management practices or addressing climate change impacts.

**Table 6:** Variables for Multiple Correspondence Analysis (MCA) in Guinea fowl production

Variable	Codes	Modalities
Survey town	Town	Dungu
		Golinga
		Kula
Age	Age group	Younger
		Young
		Old
		Older
Marital Status	Marital stat	Divorced
		Married
		Single
		Widow(er)
Literacy level	Education	Non-formal
		Primary
		Secondary
		Tertiary
		Vocational
Occupation	Main_occup	Blacksmith
		Butcher
		Crop farming
		Livestock farming
		Mechanic
		Mixed farming
		Mobile money vendor
		Student
		Teacher
		Vulcanizer
Type of feed	Feed_type	Full feed (compound feed) + supplements
		Supplements
Supplement type	Type_Suplement	Kitchen waste
		Maize
		Millet
		Sorghum
Drinking water source	Drink_water_source	River
		Pond/Well
		TAP
Keet_Mortality rate	Keet_mortality	< 20
		> 50
		20–50
Constrains to Guinea Fowl production	Constraints to GF	Disease
		High cost of production
		High mortality
		Housing
		Nutrition
		Poor productivity of birds
		Predation
		Taming of birds
		Theft
Adult guinea fowl mortality rate	Adult Mortality	< 20
		> 50
		20–50
Knowledge of climate change effect	Climate level know	No idea
		2 (Average)
		3 (Good)
		4 (Very good)
Specific knowledge of climate change effect	Specific climate	Changes in environmental temperature
		Changes in rainfall
		No idea
Experience in guinea fowl production	Experience	13–23 years
		2–12 years
		24–34 years
		35–45 years
Number of guinea fowl per flock	Flock No	High flock
		Medium–low flock
		Small flock
		Upper medium flock
Training in guinea fowl production	Training	No
		Yes

Source (Survey 2024)

**Fig. 2:**
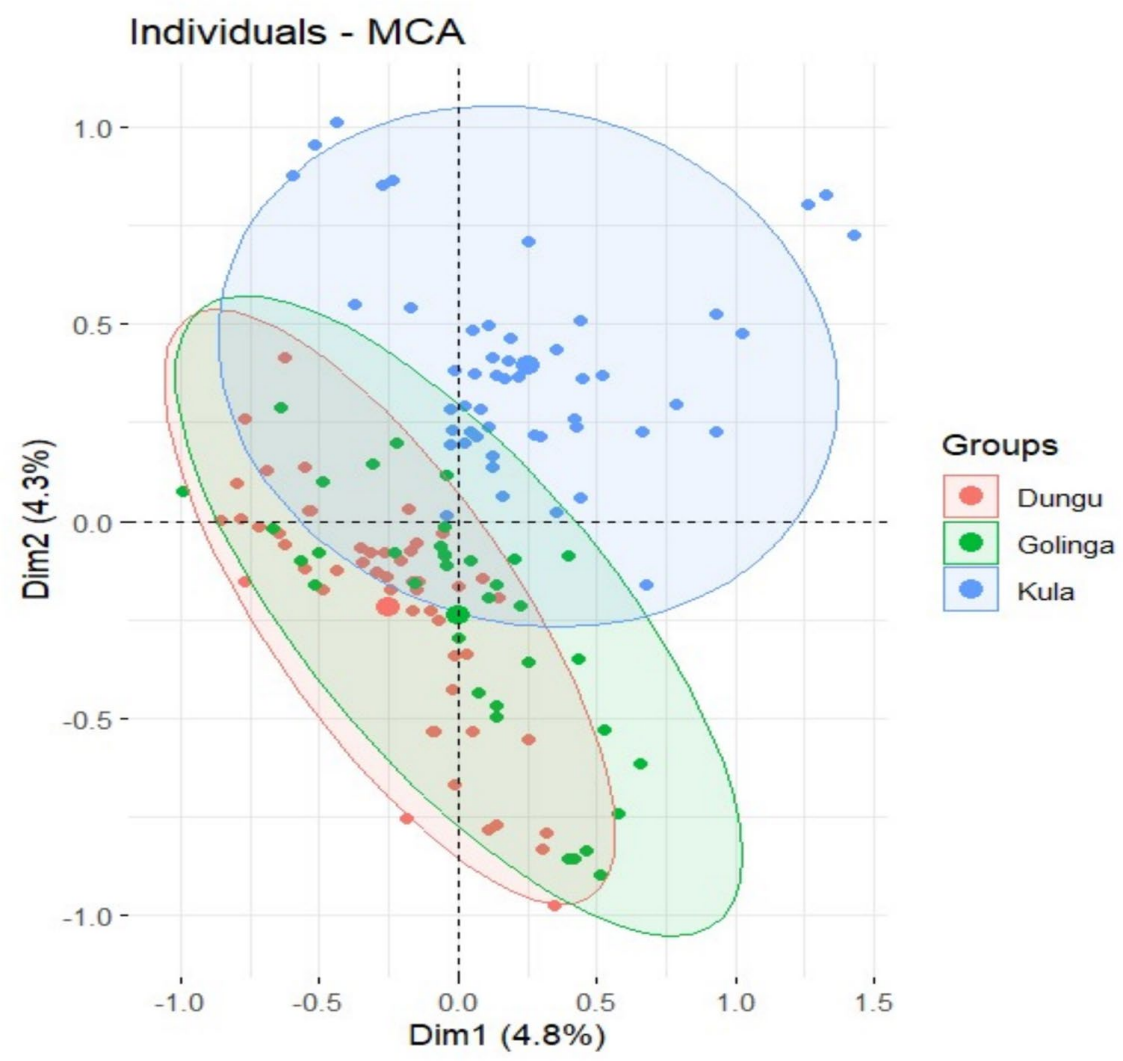
MCA mapping of the socioeconomic characteristics of the study area. *Source Author’s analysis from Survey data 2024*

**Fig. 3:**
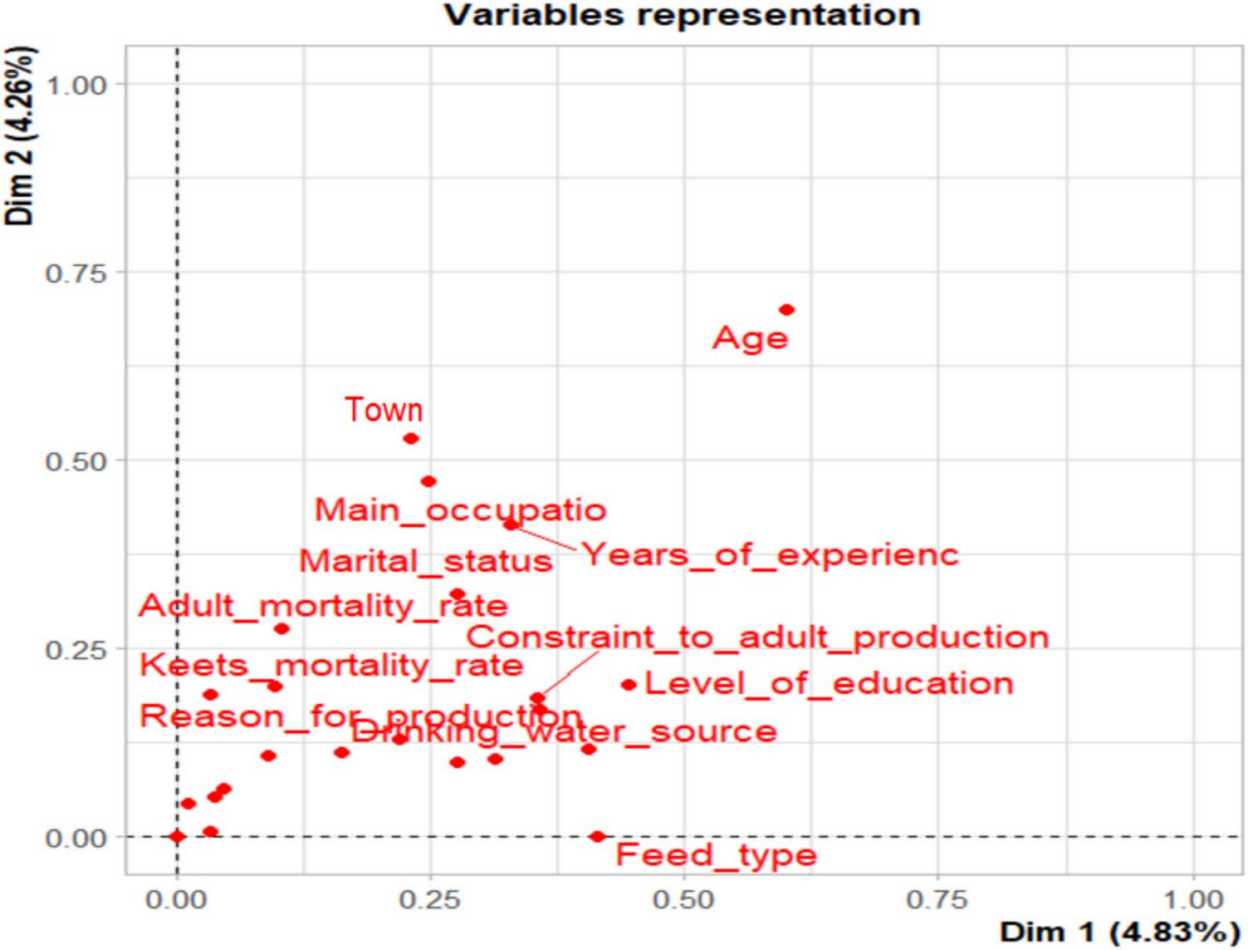
Variable MCA Plot Source (Survey 2024)

Each variable represents a key factor that could affect production outcomes, with detailed modalities capturing the diversity of responses or conditions observed in the study. The variables cover a range of socio-demographic factors (e.g., age, marital status, education, occupation), farming practices (e.g., type of feed, drinking water source, mortality rates), and contextual challenges (e.g., constraints to production, knowledge of climate change effects). This comprehensive dataset provides a robust foundation for analyzing patterns and relationships among the variables, thereby offering insights into the dynamics of guinea fowl farming systems. The MCA mapping of the socioeconomic characteristics of the study area and the variable MCA Plot Source are given in Figs. 2 and 3, respectively.

The two overlapping towns (Dungu and Golinga) likely have similar characteristics across the variables in the MCA. This means that for the dimensions captured by the MCA (which are combinations of the categorical variables), the responses or patterns from these two towns are more alike compared to the third town. This implies that the two overlapping towns may share common socio-economic, cultural, or environmental factors that influence the production variables measured.

The town (Kula) that does not overlap with the others likely differs in its production and socioeconomic profile. This town is positioned farther apart in the MCA plot, suggesting that its patterns of responses to the variables in this analysis are distinct. Implying that the town has different agricultural practices, a different approach to climate change, or dissimilar socio-economic characteristics compared to the other two. This could also reflect differences in factors like income sources, training in guinea fowl production, or constraints faced in production.

## Discussion

In this study, the age of the farmers varied significantly across the villages, with the majority of farmers in all towns falling within the 18 to 55-year range. This variation in age could be attributed to differences in social life patterns, culture, lifestyle, work, and interests among the inhabitants of the surveyed towns. Consistent with our findings, Orounladji et al. (2022) identified significant differences in the age of guinea fowl farmers across various regions of Benin (Alibori, Atacora, Atlantique, Borgou, Collines, Couffo, Donga, Mono, Plateau, and Zou), with ages ranging between 41 and 49 years. Similarly, Kouassi et al. (2019) reported that most guinea fowl farmers in the Folon, Kabadougou, Bounkani, Indénié-Djuablin, Nawa, Grands-Ponts, and Tonkpi regions of Ivory Coast were between 18 and 59 years old. In contrast, Teye and Adam (2000) found that the ages of guinea fowl farmers in the Damango area of Ghana ranged from 20 to 80 years, with the majority being between 41 and 60 years old. The minimum age of guinea fowl farmers in the Tolon and Busila North districts of Ghana was reported as 21 years, with most farmers falling within the 41 to 50-year age range (Issaka and Yeboah 2016). Similarly, Zvakare et al. (2018) found that the ages of guinea fowl farmers in the Chipinge district of Zimbabwe ranged from 27 to 83 years. In contrast, Soara et al. (2020) observed no significant difference in the ages of guinea fowl farmers between the Atakora and dry Savannah regions of Togo.

Marital status also varied significantly among the towns; however, the majority of farmers in each town were married. This trend may be attributed to the limited career opportunities in rural areas of northern Ghana, which often lead to early marriages among the youth. A study by Ahinkorah et al. (2024) revealed that women in the North East (38.2%), Western North (36.7%), and Ahafo regions (35.8%) had the highest prevalence of early marriage, while women in the Western (22.2%) and Greater Accra (19.7%) regions had the lowest prevalence in Ghana. Furthermore, Amoako Johnson et al. (2019) found higher marriage rates in districts of Northern Ghana. Although not statistically significant, Soara et al. (2020) observed a higher proportion of married guinea fowl farmers compared to unmarried farmers in two regions of Togo. Similar findings have been reported by other authors in various parts of Africa (Zvakare et al. 2018; Kouassi et al. 2019; Ahaotu et al. 2018).

The educational status of the farmers varied significantly, with the majority having non-formal educational backgrounds. This trend can be attributed to the lack of interest in formal education among inhabitants of rural northern Ghana, driven by poor educational infrastructure and the economic challenges faced by parents. Addy (2013) noted that school attendance in the Northern regions of Ghana was severely affected by poor educational management and poverty. Additionally, from a young age, most children in rural northern Ghana engage in farming activities with their parents to contribute to the family’s primary source of income. Consistent with the findings of the current study, Teye and Adam (2000) reported a higher proportion of illiterate guinea fowl farmers in the Damango area of Ghana. Similarly, Issaka and Yeboah (2016) found that most guinea fowl farmers in the Tolon and Busila North districts of Ghana had no formal education. Kouassi et al. (2019) also reported comparable findings in various regions of Ivory Coast. In contrast, Soara et al. (2020) found no significant difference in the educational backgrounds of guinea fowl farmers between the Atakora and dry Savannah regions of Togo. Meanwhile, Zvakare et al. (2018) identified a higher percentage of guinea fowl farmers with secondary education in Zimbabwe.

In the present study, the main occupation of the farmers varied significantly among the towns, with most farmers in each town being crop farmers. This observation aligns with the fact that crop farming is the primary occupation and source of livelihood for rural inhabitants in the Tolon and Tamale districts. Bawa (2019) revealed that 97.9% of the inhabitants of northern Ghana are engaged in crop farming, with only a small proportion practicing livestock farming. Consistent with our findings, Massawa et al. (2020) also identified farming as the primary activity among guinea fowl farmers in Cameroon. Similarly, Soara et al. (2020) reported no significant difference in the main occupation of guinea fowl farmers in Togo; however, the percentage of respondents engaged in crop farming as their primary occupation was the highest.

Experience in breeding guinea fowls varied significantly among the towns. This variation could be attributed to factors such as the primary occupation, age, and the level of interest of the farmers in guinea fowl production. Consistent with the findings of the present study, Orounladji et al. (2022) and Soara et al. (2020) also identified significant differences in breeding experience among guinea fowl farmers in different regions of Benin and Togo, respectively.

Although training in guinea fowl breeding did not differ significantly among the towns, most farmers had received training in breeding guinea fowls. This contrasts with the findings of Soara et al. (2020), who reported that a higher proportion of guinea fowl farmers in Togo had no training in guinea fowl breeding compared to those who were trained. Similarly, Saina (2005) found that most farmers in the Guruve district of Zimbabwe had no training in guinea fowl breeding.

In the present study, none of the guinea fowl farmers received external funding from the government or NGOs. Guinea fowl production is gradually rising in Ghana; however, the higher interest in broiler and layer hen production has overshadowed guinea fowl production due to the higher demand for chicken meat and eggs. This has caused the government to allocate more resources to broiler chicken and layer hen farmers. The lack of external funds could also be attributed to the fact that the majority of smallholder guinea fowl farmers have other businesses as their primary source of income, and therefore might not meet the criteria for obtaining a loan. In agreement with the results of the present study, Kouassi et al. (2019) also reported that most guinea fowl farmers in Ivory Coast self-financed their guinea fowl farms.

Most farmers reared their guinea fowls for both home consumption and sale, although there were no significant differences among the towns. Guinea fowl is identified as a source of protein and income for rural farmers in Northern Ghana and other parts of Africa (GreenViews 2024; Kouassi et al. 2019). In line with the findings of the current study, Massawa et al. (2020) also reported that the majority of guinea fowl farmers in Cameroon reared their birds for both home consumption and sale.

Most farmers earned more income from selling guinea fowl eggs than from selling live guinea fowls, likely due to the high demand for guinea fowl hatching and table eggs. Guinea fowl eggs are rich in protein and essential amino acids, which has gradually increased their demand, purchasing, and consumption. Additionally, the poor hatching traits of guinea fowl eggs contribute to the higher price of fertile or hatching guinea fowl eggs compared to chicken eggs. These factors likely explain why most farmers generated higher revenue from selling guinea fowl eggs than from selling live guinea fowls.

The significant variation in the prices of keets, young and adult guinea fowls, and fertile eggs across the towns may be attributed to the livestock sector's inability to regulate guinea fowl prices, largely due to the neglect of the guinea fowl business. Furthermore, this price variation could be influenced by the types of investments made by farmers during the production period, such as transportation, feed, and medicine.

Most farmers sold their birds and eggs both at home and in the market. In Dungu, Golinga, and Kula, most guinea fowl farmers raised their birds at home, and buyers typically had to locate and meet the farmers at their homes. However, during market days, when neighboring villages gather at a central market, farmers often bring their birds, along with other crops, to sell.

The significant variation in flock sizes among towns may be attributed to factors such as the level of skills and experience, the purpose of production (sale or home consumption), and the availability of resources (e.g., feed, housing). Abdul-Rahman and Adu (2017) similarly observed variations in flock sizes among guinea fowl farmers in the Tolon and Busila North districts. Other studies have also reported differences in flock sizes among guinea fowl farmers across various African countries (Massawa et al. 2020; Boko et al. 2011; Soara et al. 2020).

The Lavender breed was the most common guinea fowl breed reared by most farmers, as it was considered the most prolific for both egg and meat production. This finding contrasts with Abdul-Rahman and Adu's (2017) report that the Pearl guinea fowl breed is the most common in northern Ghana. It is possible that the Lavender breed is better adapted to the environmental conditions of the study area, giving it superior performance traits over other breeds and increasing its popularity among farmers. Onunkwo and Okoro (2015) also identified a higher egg-laying rate and hen-day egg production in Lavender guinea fowls compared to other breeds. Additionally, the higher proportion of adult guinea fowls in the flock may be attributed to marketing and breeding purposes. Similarly, Boko et al. (2011) reported that more than half of the flock in northeast Benin consisted of adult male and female guinea fowls.

A significant variation in production methods was observed among the towns, with most farmers practicing semi-intensive production. This variation may be influenced by factors such as the level of experience or the availability of resources (e.g., finance, feed, water, and housing). Similarly, Abdul-Rahman and Adu (2017) found that all farmers in their study in the Tolon district practiced semi-intensive production. In the Atakora and dry savannah regions of Togo, production methods also varied significantly, with semi-intensive production being the most common (Soara et al. 2020). Significant variations in production methods have also been reported among guinea fowl farmers in different regions of Benin (Orounladji et al. 2022). However, our findings contrast with those of Kouassi et al. (2019) and Gono et al. (2013), who reported a higher prevalence of extensive systems among guinea fowl farmers in Ivory Coast and Zimbabwe, respectively.

In this study, a significant variation in housing structures was found among the towns, with most farmers using traditional mud houses, which may reflect the financial background and available resources of the farmers. Our findings are consistent with Abdul-Rahman and Adu (2017), who also reported that most guinea fowl farmers in the Tolon district used mud houses. Kusina et al. (2012) reported that guinea fowls in Zimbabwe were housed in poor structures, and Massawa et al. (2020) identified similar housing materials (cotton, sorghum stalks, and red millet covered with straw) in Cameroon. Similarly, Boko et al. (2011) observed that mud houses were used for housing guinea fowls at night in Benin. Zvakare et al. (2018) also reported that most guinea fowl shelters in Zimbabwe were made from local materials.

The significant variation in feed types among the towns may be attributed to factors such as the farmer’s objectives, experience, education, financial status, and primary occupation. The majority of farmers in Dungu and Golinga used commercial guinea fowl feed, while the majority in Kula used both broiler and layer chicken feed, possibly influenced by feed availability, price, and performance outcomes.

Most farmers supplemented their birds'diets with maize, millet, or sorghum, which may be related to the availability of feed resources, prices, educational background, previous experience, and the farmers'primary occupation. Consistent with the present study, Issaka and Yeboah (2016) reported that in Tolon and Busila North districts of Ghana, keets were fed mainly sorghum grit or ground maize, while adult guinea fowls were fed with maize or sorghum grains. In Togo, 90.2% and 64.6% of farmers supplemented their young guinea fowls with maize and sorghum, respectively (Soara et al. 2020). Zvakare et al. (2018) also reported that in Zimbabwe, 45.2% of guinea fowl farmers supplemented their birds with maize and sorghum. Cereals (maize, millet, sorghum, and rice) were identified as the main feed for guinea fowls in other African countries (Lengthang et al. 2023; Kusina et al. 2012; Massawa et al. 2020; Ahaotu et al. 2018).

All the guinea fowl farmers provided drinking water to their birds either from ponds or rivers. This may be due to the harsh climatic conditions in northern Ghana, which often lead to droughts, resulting in the death of livestock and plant species. Additionally, because of the lack of potable water at homes, ponds, rivers, and wells are the primary sources of drinking water for most rural inhabitants of northern Ghana. In fact, it was reported that 60–70% of households in northern Ghana rely on boreholes for drinking water (Shier et al. 1996), and Saana et al. (2016) noted that boreholes draw water from streams and rivers. In addition, natural water bodies were identified as the main source of drinking water in rural communities in northern Ghana (Cobbina et al. 2015).

In the study areas, wing drooping was the main symptom of disease, and hygiene plus vaccination were the primary health management practices, with the majority of farmers vaccinating their birds once a year. In agreement with our findings, Soara et al. (2020) also identified that the majority of guinea fowl farmers in the Atakora and Dry Savannah regions of northern Togo practiced hygiene plus vaccination as a disease prevention technique, with wing drooping identified as the main symptom of guinea fowl disease. However, Lombo et al. (2018) reported that 23.58% of farmers in northern Togo do not vaccinate their guinea fowls, with 62.26% vaccinating their birds twice a year.

The majority of farmers in all the towns vaccinated their birds themselves, using both modern and traditional treatments. Soara et al. (2020) also reported that the use of modern and traditional treatments was the highest among farmers in the Atakora and Dry Savannah regions of northern Togo. However, vaccination of guinea fowls was mostly practiced by a combination of veterinarians and farmers. Zvakare et al. (2018) reported that although none of the guinea fowl farmers in Zimbabwe vaccinated their birds, both ethno-veterinary and conventional treatment techniques were used during disease outbreaks.

High mortality was the main constraint to keet survivability, with mortality rates exceeding 50% in all the towns. Previous surveys in the Tolon district and Damango area of Ghana also identified high mortality as the major constraint to keet production (Teye and Adam 2000; Abdul-Rahman and Adu 2017). In the Tolon and Busila North districts of Ghana, between 32 and 34 keets are lost annually per farmer (Issaka and Yeboah 2016). Additionally, 49.1% of guinea fowl farmers in Benin reported higher mortality in birds between 0 and 6 weeks of age (Orounladji et al. 2022). A study by Kouassi et al. (2019) revealed that 95% of guinea fowl farmers in Ivory Coast complained about high keet mortality. Other authors in different African countries also reported high keet mortality as the major hindrance to guinea fowl production (Ahaotu et al. 2018; Boko et al. 2011). However, predation was identified as the major constraint to keet production in Togo (Soara et al. 2020).

While taming of birds and predation were the major constraints to adult guinea fowl production, the mortality rate of adult guinea fowls varied between < 20% and 50% in the survey area. Indeed, Abdul-Rahman and Adu (2017) also identified predation as the second most important constraint to guinea fowl production in the Tolon district of Ghana. In Zimbabwe, Zvakare et al. (2018) identified predation by snakes, dogs, and cats as the major constraint to guinea fowl production. Moreover, predation was reported as the second most important constraint to guinea fowl production in Chad and Togo (Lengthang et al. 2023; Soara et al. 2020).

All the farmers in the current study were aware of climate change, with the majority in all towns having"good"knowledge about climate change. This could be associated with rapid changes in environmental conditions due to global warming over the past decade. Changes in environmental temperature were identified as the most common sign of climate change by the majority of farmers. Indeed, Adedeji et al. (2014) identified changes in ambient temperature as evidence of climate change.

In addition, the rapid spread of diseases was considered the most profound effect of climate change on guinea fowl production, with climate change-related mortality varying between < 20% and 50%. Patterson and Guerin (2013) reported that climate change could affect vector, pathogen, and reservoir ecology, potentially altering disease transmission intensity, range limits, and the emergence and re-emergence of pathogens affecting commercial poultry. Additionally, climate change was identified as a potential factor for the outbreak of H5 N1, as it can change transmission pathways (Mu et al. 2014).

The majority of farmers reported that climate change led to additional costs in disease prevention. Over the past decade, rapid emergence, re-emergence, and spread of poultry diseases have caused a devastating blow to the poultry industry due to high mortality, increasing the overall cost of disease and health management.

The results of the Multiple Correspondence Analysis (MCA) offer significant insights into the patterns of individual and variable relationships across three distinct groups: Dungu, Golinga, and Kula. These insights provide valuable implications for understanding production practices, household characteristics, and resource access in the regions under study.

The individual MCA plot reveals the grouping of individuals into three categories, represented as Dungu, Golinga, and Kula. The ellipses for Dungu (red) and Golinga (green) overlap considerably, suggesting similarities in production practices or household characteristics between these two towns. This overlap may indicate shared economic constraints, common production techniques, or environmental conditions. In contrast, the blue points for Kula are more dispersed, reflecting greater variability in practices or characteristics within this group. This dispersion could imply diverse challenges or innovations that differentiate Kula from the other regions.

The dimensions provide additional insights into the variability within the data. Dim1, which explains 4.8% of the variability, likely reflects factors related to production efficiency, such as feed type or access to resources. Dim2, accounting for 4.3% of the variability, appears to represent secondary factors like socio-demographic attributes, including age or education level. Notably, the clustering of Dungu and Golinga highlights their shared conditions, while Kula's distinct clustering underscores the need to investigate the unique aspects influencing this group.

The variable MCA plot highlights the contributions of specific variables to the observed variability. Variables such as Feed_type are strongly associated with Dim1, indicating their critical role in explaining the variability related to production factors. On the other hand, Age stands out as a key contributor to Dim2, suggesting its significance in differentiating individuals based on socio-demographic characteristics rather than production-related factors.

The interconnectedness of variables such as Town, Main_occupation, Years_of_experience, and Level_of_education suggest that these factors collectively explain similar patterns in the data. Their close clustering indicates a potential overlap in their influence on production practices or household characteristics. Interestingly, the positioning of Feed_type near the origin suggests its balanced contribution across both dimensions, possibly linking socio-economic and production-related factors.

Outliers, such as the variable Age, are positioned far from the other variables, highlighting their unique contribution to the variability. This outlier position could reflect generational differences in farming practices or resource access, making age a critical factor to consider in policy and program development.

The clustering patterns observed in the MCA plots emphasize shared production constraints in Dungu and Golinga, such as challenges related to feed and water access. These shared conditions highlight the need for region-specific interventions to address these common issues. Conversely, the dispersion of data points for Kula suggests that this region may face distinct challenges or exhibit diverse practices, necessitating targeted interventions that address its unique circumstances.

The prominence of Age as a critical variable underscores the importance of designing age-specific interventions. For instance, younger farmers could be encouraged to adopt innovative practices through training and access to modern technologies, while older farmers might benefit from adaptive resources and support tailored to their specific needs. Recognizing the diverse needs of different age groups can enhance the overall effectiveness of development programs.

The insights gained from the MCA align with the United Nations Sustainable Development Goals (SDGs), particularly those related to zero hunger and poverty reduction. Addressing factors such as feed type, education, and experience can boost productivity and resilience in the region. Tailored programs focusing on these factors can contribute to sustainable agricultural development and improve livelihoods.

## Conclusion

Our findings revealed significant guinea fowl production in the study area. Although indigenous guinea fowl farming is primarily a secondary activity with low productivity, it plays a crucial socio-economic role—providing income and nutrition—for rural families, warranting special attention. Guinea fowl production in the surveyed area is predominantly carried out by illiterate small- and medium-scale holders who lack financial support. Housing and management practices for guinea fowls are largely suboptimal in the study area. This study also highlighted major constraints to guinea fowl production, including high keet mortality, difficulties in taming birds, predation, and a lack of access to qualified veterinarians. Additionally, the increasing prevalence of pests and diseases, exacerbated by climate change, has negatively impacted the income generated from guinea fowl farming.

To enhance guinea fowl production in the study area, policy interventions should focus on improving farmers'access to knowledge and resources for effective disease management and climate resilience. This includes promoting research into the phenotypic, genetic, and molecular characteristics of indigenous guinea fowl breeds to enhance their growth, disease resistance, and overall welfare. Furthermore, strengthening agricultural extension services and providing targeted training on modern farming techniques, disease prevention, and climate adaptation strategies would empower farmers to overcome production challenges and improve the sustainability of guinea fowl farming.

## Data Availability

The data for this research is available with the corresponding author and will be shared upon request.
